# Diagnosis of extrapulmonary tuberculosis by ultrasound-guided biopsy: A retrospective comparison study

**DOI:** 10.3389/fcimb.2023.1154939

**Published:** 2023-03-22

**Authors:** Jin-Chuan Xu, Xia Shi, Xin Ma, Wen-fei Gu, Zhi-xiong Fang, Hui Zhang, Xiao-Yong Fan

**Affiliations:** ^1^ Shanghai Public Health Clinical Center, Fudan University, Shanghai, China; ^2^ School of Laboratory Medicine and Life Science, Wenzhou Medical University, Wenzhou, China; ^3^ Department of Infectious Diseases and Public Health, Central Hospital of Xiangtan, Xiangtan, China

**Keywords:** extrapulmonary tuberculosis (EPTB), diagnosis, biopsy, Xpert, human immunodeficiency virus (HIV)

## Abstract

**Objective:**

To compare the diagnostic performance of laboratory assays on the ultrasound-guided core needle biopsy samples for diagnosis of extra-pulmonary tuberculosis (EPTB) in HIV-positive and HIV-negative patients.

**Methods:**

A total of 217 patients suspected to have EPTB underwent lesion biopsy from 2017 to 2020. Results of laboratory tests on the biopsy and non-biopsy samples were collected with clinical data for retrospective analysis of test utility. The calculated diagnostic accuracy of the tests was stratified according to the specimen types and HIV status.

**Results:**

The cohort contained 118 patients with a final positive diagnosis of extrapulmonary tuberculosis (EPTB group, 54.4%) and 99 finally diagnosed as without TB (non-EPTB group, 45.6%). The risk factor for EPTB was HIV co-infection (OR 2.22, 95% CI 1.17-4.28, *p* = 0.014). In biopsy samples, GeneXpert (Xpert) showed higher sensitivity (96.6% [91.6-98.7], *p* < 0.0001) than culture (56.1% [47.0-64.9]). Regardless of HIV status, Xpert had the highest sensitivity (>95%) and specificity (nearly 100%) of any methods. In non-biopsy samples, only T-SPOT.*TB* (T-SPOT) showed higher sensitivity than culture (90.9% [62.3-99.5] *vs* 35.3% [17.3-58.7], *p* = 0.0037). Furthermore, the sensitivities of Xpert were lower in non-biopsy samples (60.0% [23.1-92.9], *p* = 0.022) than in biopsy samples (100% [86.7-100]). Even in smear-negative biopsy samples, Xpert still had higher sensitivity than culture and retained high specificity (100% [95.7-100]).

**Conclusion:**

Superior performance of Xpert in diagnosing EPTB was observed regardless of HIV status and specimen types. Nevertheless, the biopsy samples still substantially facilitated the accurate diagnosis of extrapulmonary tuberculosis.

## 1 Introduction

Tuberculosis (TB) is a communicable disease caused by the mycobacterium tuberculosis complex (MTBC). Globally, an estimated 10.6 million people (range, 9.9-11.0 million) fell ill with TB in 2021, an increase of 4.5% from 10.1 million (95% UI: 9.5-10.7 million) in 2020; and about 1.6 million died from TB in the same year, up from a best estimate of 1.5 million in 2020 (WHO, 2022).

Extrapulmonary tuberculosis (EPTB) refers to TB occurring in parts of the body other than the lungs (e.g., lymph nodes, meninges, abdomen, pleura, genitourinary tract, skin, joints, and bones) (Golden and Vikram, 2005). As per the Global TB Report 2020, EPTB constituted 16% of the 7.5 million notified TB cases in 2019, ranging from 8% in the Western Pacific Region to 24% in the Eastern Mediterranean Region (WHO, 2020). In China, EPTB accounted for approximately 24% of TB cases, with a maximum of 33% in the western region (Li et al., 2022). In the context of WHO’s End TB Strategy, timely diagnosis and treatment of EPTB is a challenge we have to face.

The main risk factors associated with EPTB vary widely and include human immunodeficiency virus (HIV) co-infection, female sex, age (young children or over 65 years of age), and diabetes (Shivakoti et al., 2017; Ohene et al., 2019; Pang et al., 2019; Banta et al., 2020). Due to the absence of typical TB symptoms, EPTB is often misdiagnosed as other diseases, such as cancers (Xiang et al., 2021) and inflammatory diseases (Aisenberg et al., 2005; Jain, 2011). Laboratory diagnosis plays a decisive role in the diagnosis of EPTB. However, studies comparing various laboratory assays based on biopsy samples are limited, probably because biopsy samples are not readily available (Norbis et al., 2014; Park and Kon, 2021).

This study analyzed the records from laboratory investigations of specimens from suspected extrapulmonary tuberculosis patients in an infectious disease hospital from 2017 to 2020 to compare the accuracy of different methods of laboratory diagnosis.

## 2 Methods

### 2.1 Study population and specimens

This study was conducted in Shanghai Public Health Clinical Center, one of the designated National Tuberculosis Hospitals in China. Patients with suspected EPTB (WHO, 2021a) who had undergone biopsy between July 01 2017 and September 30 2020 were enrolled. The inclusion criteria were patients with lymph node enlargement and typical symptoms of TB (fever, wasting, night sweats, etc.), or a positive PPD/TSPOT.TB test, or suspicion of TB on imaging, and willing to receive puncture procedures. The exclusion criteria were the patient refusing the biopsy or patients with contraindications to puncture, such as coagulation dysfunction. The biopsy samples were collected by an ultrasound-guided core needle biopsy. For the non-biopsy samples, we collected data from the hospital’s Laboratory Examination Control System by matching the patient’s ID and the exact test date. Demographic information (sex, age, HIV status, and diagnosis) and anatomical locations of EPTB were recorded upon enrollment. The results of pathological and microbiological tests were included.

### 2.2 Clinical definition and classification

The culture (combined with the MPB64 test) and Xpert results were used as a microbiological reference standard. Patients were eventually classified into the EPTB group (culture (4 cases), Xpert (54 cases), or culture-Xpert (60 cases) positive) and non-EPTB group [culture and Xpert negative (99 cases)].

### 2.3 Laboratory methods

Biopsy samples were collected by ultrasound-guided biopsy in the Ultrasound Intervention Department and sent to the Laboratory and Pathology Departments for diagnostic tests and histological examinations. Non-biopsy samples were collected and tested routinely in the Laboratory Department. An optimized sample pre-treatment process was used to concentrate mycobacteria in the specimens and thus improve the accuracy of the assays (Rickman and Moyer, 1980; Peterson et al., 1999). Briefly, Large-volume liquid specimens were first centrifuged at 3000-3800g for 15 min, the supernatant was discarded and digested with 2-4% NaOH for 15-20 min. Solid samples were digested directly with 2-4% NaOH. After digestion, the samples were neutralized with sterile PBS, then centrifuged at 3000-3800g for 15 min and the supernatant was discarded. The digested samples were mixed with 0.1-1mL of PBS and used for subsequent assays. Routine tests included culture (BACTEC MGIT 960 rapid culture method), smear (Auramine O staining kit, Zhuhai Baso Biotechnology Co.), and Xpert (Gene X-Pert MTB/RIF, Cepheid, USA). T-SPOT.TB (Oxford Immunotec Ltd, UK), was carried out using kits based upon the hospital’s programmatic laboratory procedures. Species identification was carried out with an MPB64 monoclonal antibody assay (Hangzhou Genesis Biodetection & Biocontrol Co., Ltd, Hangzhou, China) based on positive cultures. Next-generation sequencing is done by Shanghai Simple Gene Medical Laboratory (Kindstar Globalgene Technology, Inc. Shanghai, China) when required.

The pathological tissues were fixed with 4.0% formaldehyde, routinely dehydrated and paraffin-embedded, and serially sectioned at a thickness of 4 μm. HE stain and acid-fast stain (Zhuhai Baso Biotechnology Co. Zhuhai, China) were performed in sections for routine microscopic diagnosis. EPTB positive was identified when there was typical epithelioid granuloma formation, caseation, and positive acid-fast staining.

### 2.4 Statistical analysis

We used R studio version 4.0.0 to process the data and GraphPad Prism version 8.0 for all analyses. The baseline table was performed using the R‐based tableone package (version 0.13.2). The χ² test (including McNemar’s test) was used to calculate differences in diagnostic accuracy metrics; the Mann-Whitney U test was used to calculate differences in non-parametric data; the two-sample proportion test (Chi-square test) was used to compare, for example, sensitivity across two groups.

## 3 Results

**Figure 1:**
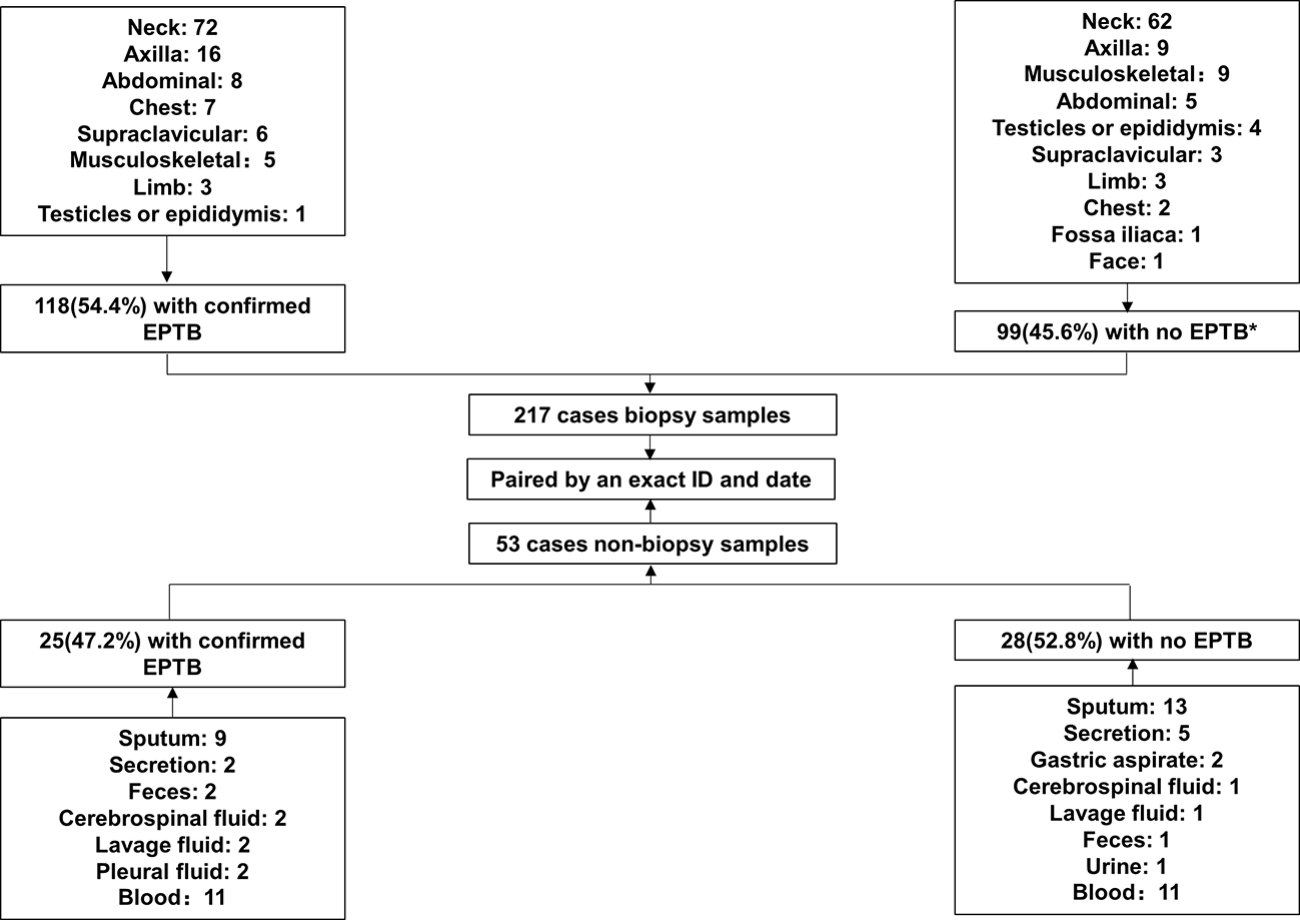
Study profile.* 26 cases of lymphadenitis, 2 cases of NTM infection, 3 cases of BCG infection, 8 cases of tumor, 4 cases of *Penicillium marneffei* infection, 3 cases of *Staphylococcus aureus* infection, and 53 cases of other non-TB diseases.

**Figure 2:**
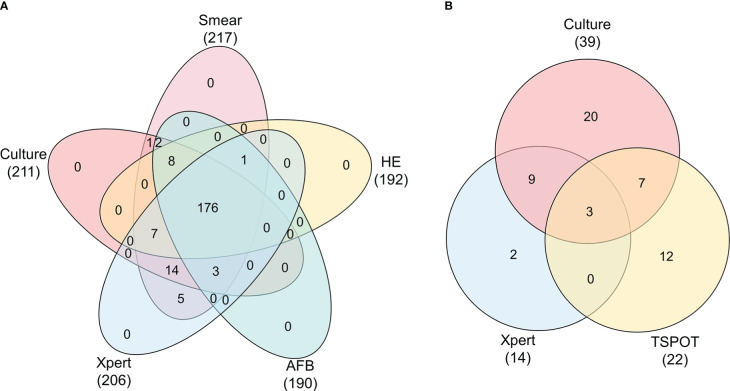
Venn diagram showing the relationship between tests of biopsy and non-biopsy samples. Data are shown stratified according to: **(A)** Veen diagram of culture, smear, HE, AFB, Xpert tests in biopsy samples, **(B)** Veen diagram of culture, Xpert, TSPOT tests in non-biopsy samples.

In this study, we enrolled 217 cases of suspected EPTB, including 118 (54.4%) cases that had been confirmed as EPTB patients and 99 (45.6%) cases that had been finally diagnosed as non-EPTB patients (**Figure 1**). The locations of the biopsy were the neck (134 cases), axillary (25 cases), musculoskeletal (14 cases), abdominal (13 cases), chest (9 cases), supraclavicular (9 cases), limb (6 cases), testicular or epididymal (5 cases), fossailiaca (1 case) and face (1 case). In addition, data from 53 non-biopsy samples (mostly sputum) were retrieved based on patient ID and sampling date. As shown in **Figure 2**, culture, smear, Xpert, Hematoxylin-eosin staining (HE), and Acid Fast Bacteria (AFB) Stain were performed on biopsy samples. For non-biopsy samples, culture, Xpert, and TSPOT assays were done.

Males were more likely to have EPTB than females (OR 1.89, 95%CI 1.10-3.29, *p* = 0.021). Using patients < 25 years of age as a control group, we found that patients exhibited an increased risk of extrapulmonary TB with increasing age (OR 1.16, 95% CI 0.60-2.25 for patients 25-44 years of age; OR 1.30, 95% CI 0.58-2.93 for patients 45-64 years of age; OR 2.05, 95% CI 0.76-5.76 for patients > 65 years of age). As expected, HIV-positive patients were more frequently affected by EPTB than HIV-negative ones (OR 2.22, 95% CI 1.17-4.28, *p* = 0.014). However, the lump diameter, pus volume, and length of patients’ biopsy samples were not related to the likelihood of a positive diagnosis of EPTB (**Table 1**).

**Table 1:** Demographic and clinical characteristics of the studied patients*.

Characteristic	Level	Overall	EPTB	Non-EPTB	OR	*P* Ratio
		217	118	99		
Gender (%)	Female	102 (47.0)	64 (54.2)	38 (38.4)	Ref.	Ref.
	Male	115 (53.0)	54 (45.8)	61 (61.6)	**1.89 [1.10;3.29]**	**0.021**
Age (%)	< 25	61 (28.1)	36 (30.5)	25 (25.3)	Ref.	Ref.
	25-44	94 (43.3)	52 (44.1)	42 (42.4)	1.16 [0.60;2.25]	0.655
	45-64	40 (18.4)	21 (17.8)	19 (19.2)	1.30 [0.58;2.93]	0.527
	> 65	22 (10.1)	9 (7.6)	13 (13.1)	2.05 [0.76;5.76]	0.156
HIV (%)	Negative	166 (76.5)	98 (83.1)	68 (68.7)	Ref.	Ref.
	Positive	51 (23.5)	20 (16.9)	31 (31.3)	**2.22 [1.17;4.28]**	**0.014**
LD (median [IQR])		28.0 [21.0, 41.7]	30.0 [23.0, 42.0]	25.0 [20.0, 40.0]	0.98 [0.96;1.01]	0.179
PV (median [IQR])		0.0 [0.0, 1.5]	0.0 [0.0, 2.75]	0.0 [0.0, 0.5]	1.01 [0.99;1.02]	0.607
SL (median [IQR])		20.0 [10.0, 30.0]	20.0 [10.0, 30.0]	20.0 [11.0, 25.0]	1.0 [0.99;1.01]	0.798

*LD, Lump diameter (mm); PV, Pus volume (ml); SL, Sample length (mm); Ref, Reference variable in categorical variables; OR, Odds ratio.

Bold means the P value is less than 0.05, with a statistical difference.

Firstly, we compared the diagnostic accuracy of conventional assays with 217 biopsy samples (**Table 2**). In biopsy samples, the sensitivity, specificity, positive predictive value (PPV), and negative predictive value (NPV) of the culture were56.1% [47.0-64.9], 96.9% [91.3-99.2], 95.5% [87.6-98.8] and 57.2% [32.6-72.6], respectively. Notably, Xpert had higher sensitivity (96.6% [91.6-98.7] vs 56.1% [47.0-64.9]; *p* < 0.0001), specificity (100% [95.8-100] vs 96.9% [91.3-99.2]; *p* = 0.096, and PPV (100% [96.7-100] vs 95.5% [87.6-98.8]; *p* = 0.023), and NPV (95.7% [89.4-98.3] vs 57.2% [32.6-72.6]; *p* < 0.0001) when compared with culture (**Table 2**). The histological assays (HE and AFB) had a better sensitivity performance (HE 92.4% [85.7-96.1], *p* < 0.0001; AFB 81.7% [73.2-88.0], *p* < 0.0001) and NPV (HE 88.6% [79.0-94.1], *p* = 0.0003; AFB 76.8% [66.6-84.6], *p* = 0.07) than culture, but poorer performance in specificity (HE 71.3% [61.0-79.7], *p* < 0.0001; AFB 73.3% [63.1-81.5], *p* < 0.0001), and PPV (HE 79.5% [71.5-85.7], *p* = 0.003; AFB 78.7% [70.1-85.4], *p* = 0.0024), consistent with a previous report (sensitivity: 95.6%, specificity: 64.6%, PPV: 74.1%, NPV: 93.2%)(Bennani et al., 2019). Unexpectedly, the sensitivity and NPV of the smear were slightly higher than that of the culture, but the difference was not significant (**Table 2**).

**Table 2:** Diagnostic utility of culture, smear, Xpert, HE, and AFB in the examination of biopsy samples.

Test and (*p*)	Sensitivity	Specificity	PPV	NPV
Culture	64/114 (56.1; 47.0-64.9)	94/97 (96.9; 91.3-99.2)	64/67 (95.5; 87.6-98.8)	94/144 (57.2; 32.6-72.6)
Smear	77/118 (65.3; 56.3-73.2)	95/99 (96.0; 90.1-98.4)	77/81 (95.1; 88.0-98.1)	95/136 (69.9; 61.7-76.9)
Smear vs Culture (*p*)	0.16	0.72	0.90	0.41
Xpert	114/118 (96.6; 91.6-98.7)	88/88 (100; 95.8-100)	114/114 (100; 96.7-100)	88/92 (95.7; 89.4-98.3)
Xpert vs Culture (*p*)	**< 0.0001**	0.096	**0.023**	**< 0.0001**
HE	97/105 (92.4; 85.7-96.1)	62/87 (71.3; 61.0-79.7)	97/122 (79.5; 71.5-85.7)	62/70 (88.6; 79.0-94.1)
HE vs Culture (*p*)	**< 0.0001**	**< 0.0001**	**0.0030**	**0.0003**
AFB	85/104 (81.7; 73.2-88.0)	63/86 (73.3; 63.1-81.5)	85/108 (78.7; 70.1-85.4)	63/82 (76.8; 66.6-84.6)
AFB vs Culture (*p*)	**< 0.0001**	**< 0.0001**	**0.0024**	0.070

Shown are the fraction of positive results, n/N and % with 95% CI in parentheses, with p-values where appropriate. NPV, negative predictive value; PPV, positive predictive value.

Bold means the P value is less than 0.05, with a statistical difference.

**Figure 3:**
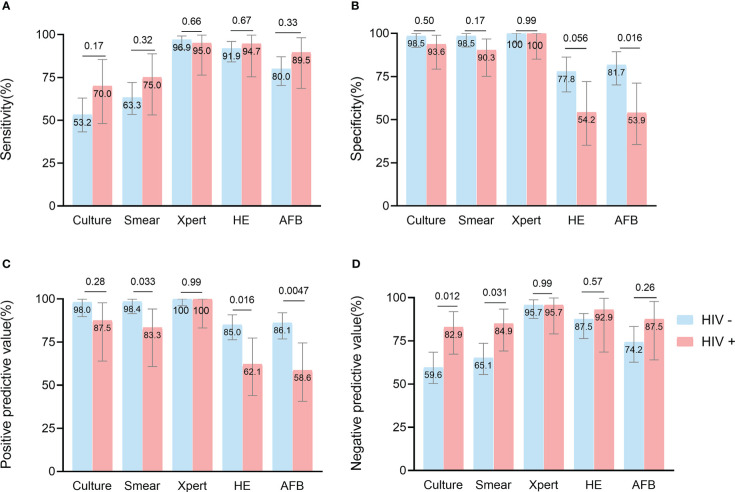
Head-to-head comparison of test accuracy in biopsy samples, by HIV status. Data are shown stratified according to: **(A)** sensitivity, **(B)** specificity, **(C)** positive predictive value, **(D)** negative predictive value.

By HIV status, culture had greater sensitivity (70.0% [48.1-85.5] vs 53.2% [43.2-63.0], *p* = 0.17) and NPV (82.9% [67.3-91.9] vs 59.6% [50.3-68.4], *p =* 0.012) in HIV-positive patients than HIV-negative ones. Both specificity (93.6% [79.3-98.9] vs 98.5% [91.9-99.9], *p* = 0.50) and PPV (87.5% [64.0-97.8] vs 98.0% [89.7-99.9], *p* = 0.28) of culture were lower in HIV-positive patients than in HIV-negative patients. Similar trends were observed for the smear and histological methods, but the specificity (HE: 54.2% [35.1-72.1] vs 77.8% [66.1-86.3], *p* = 0.056; AFB: 53.9% [35.5-71.2] vs 81.7% [70.1-89.4], *p* = 0.016) and PPV (smear: 83.3% [60.8-94.2] vs 98.4% [91.5-99.9], *p* = 0.033; HE: 62.1% [44.0-77.3] vs 85.0% [76.3-90.8], *p* = 0.016; AFB: 58.6% [40.7-74.5] vs 86.1% [76.8-92.0], *p* = 0.0047) of the assays for HIV-positive patients were significantly lower than for HIV-negative patients, because HIV-positive patients were more likely to be infected by non-tuberculous mycobacteria (NTM)(Álvaro-Meca et al., 2015) (3.8% in this study). Remarkably, Xpert had the highest sensitivity, specificity, PPV, and NPV values of all assays and did not differ significantly between HIV-positive and negative patients (**Figure 3**; **Supplementary Table S1**).

**Figure 4:**
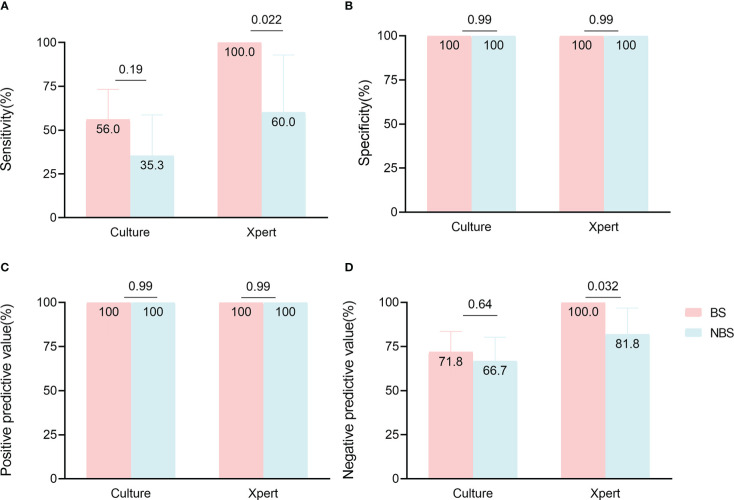
Comparison of culture and Xpert accuracy in paired biopsy samples (BS) and non-biopsy samples (NBS). Data are shown stratified according to **(A)** sensitivity, **(B)** specificity, **(C)** positive predictive value, **(D)** negative predictive value.

In 53 non-biopsy samples (**Table 3**), TSPOT had higher sensitivity than culture (90.9% [62.3-99.5] vs 35.3% [17.3-58.7], *p* = 0.0037), but lower specificity (63.6% [35.4-84.8] vs 100% [85.1-100], *p* = 0.0026). The sensitivity and specificity of Xpert and culture did not differ significantly, most likely due to the small sample size (**Table 3**). We also compared the performance of different assays between biopsy samples and non-biopsy samples. The Xpert showed higher sensitivity (100% [86.7-100] vs 60.0% [23.1-92.9], *p* = 0.022) and NPV (100% [86.2-100] vs 81.8% [52.3-96.8], *p* = 0.032) in biopsy samples than in non-biopsy samples, but not higher specificity or PPV. The same trend was observed for culture, but not significantly (**Figure 4**; **Supplementary Table S2**).

**Table 3:** The diagnostic accuracy of culture, smear, Xpert, PCR, and TSPOT in non-biopsy samples.

Test and (*p*)	Sensitivity	Specificity	PPV	NPV
Culture	6/17 (35.3; 17.3-58.7)	22/22 (100; 85.1-100)	6/6 (100; 61.0-100)	22/33 (66.7; 49.6-80.3)
Xpert	3/5 (60.0; 23.1-92.9)	9/9 (100; 70.1-100)	3/3 (100; 43.9-100)	9/11 (81.8; 52.3-96.8)
Xpert vs Culture (*p*)	0.32	> 0.99	> 0.99	0.34
TSPOT	10/11 (90.9; 62.3-99.5)	7/11 (63.6; 35.4-84.8)	10/14 (71.4; 45.4-88.3)	7/8 (87.5; 52.9-99.4)
TSPOT vs Culture (*p*)	**0.0037**	**0.0026**	0.14	0.25

Shown are the numbers of positive results, n/N and % with 95% CI in parentheses, with p-values where appropriate. NPV, negative predictive value; PPV, positive predictive value.

Bold means the P value is less than 0.05, with a statistical difference.

In smear-negative biopsy samples (**Table 4**), the Xpert had significantly higher sensitivity (92.7% [80.6-97.5] vs 45.0% [30.7-60.2], *p* < 0.0001) and NPV (96.6% [90.5-99.1] vs 80.9% [72.7-87.0], *p* = 0.0007) than culture, and comparable specificity and PPV to culture. The sensitivity (86.5% [72.0-94.1] vs 45.0% [30.7-60.2], *p* = 0.0001) and NPV (92.5% [83.7-96.8] vs 80.9% [72.7-87.0], *p* = 0.033) of HE were also higher than culture, while the specificity (HE: 73.8% [63.5-82.0] vs 100% [96.0-100], *p* < 0.0001; AFB: 75.9% [65.7-83.8] vs 100% [96.0-100], *p* < 0.0001) and PPV (HE: 59.3% [46.0-71.3] vs 100% [82.4-100], *p* = 0.0012; AFB: 52.4% [37.7-66.6] vs 100% [82.4-100], *p* = 0.0003) of histological methods were lower than culture (**Table 4**).

**Table 4:** Culture, Xpert, HE, and AFB diagnostic accuracy in smear-negative biopsy samples.

Test and (*p*)	Sensitivity	Specificity	PPV	NPV
Culture	18/40 (45.0; 30.7-60.2)	93/93 (100; 96.0-100)	18/18 (100; 82.4-100)	93/115 (80.9; 72.7-87.0)
Xpert	38/41 (92.7; 80.6-97.5)	85/85 (100; 95.7-100)	38/38 (100; 90.8-100)	85/88 (96.6; 90.5-99.1)
Xpert vs Culture (*p*)	**< 0.0001**	> 0.99	> 0.99	**0.0007**
HE	32/37 (86.5; 72.0-94.1)	62/84 (73.8; 63.5-82.0)	32/54 (59.3; 46.0-71.3)	62/67 (92.5; 83.7-96.8)
HE vs Culture (*p*)	**0.0001**	**< 0.0001**	**0.0012**	**0.033**
AFB	22/37 (59.5; 43.5-73.7)	63/83 (75.9; 65.7-83.8)	22/42 (52.4; 37.7-66.6)	63/78 (80.8; 70.7-88.0)
AFB vs Culture (*p*)	**0.20**	**< 0.0001**	**0.0003**	0.98

Shown are the numbers of positive results, n/N and % with 95% CI in parentheses, with p-values where appropriate. NPV, negative predictive value; PPV, positive predictive value.

Bold means the P value is less than 0.05, with a statistical difference.

## 4 Discussion

In this retrospective analysis, we used biopsy samples and non-biopsy samples from patients with presumptive EPTB to determine the diagnostic accuracy, sensitivity, and specificity of the assays. Our key finding was that Xpert performed better than other laboratory assays regardless of the HIV status of the patients or the types of specimens. Overall, the biopsy samples provided more realistic pictures of the patient’s conditions and a more accurate diagnosis of EPTB than non-biopsy samples.

From the demographic aspects, several studies have reported similar findings that HIV co-infection and age (> 65 years old) contribute to EPTB infection (Lakoh et al., 2020; Winter et al., 2020; Barreto-Duarte et al., 2021), consistent with our results. However, we found that males were more likely to have EPTB than females (OR 1.89, 95%CI 1.10-3.29, *p* = 0.021), which was not consistent with some previous studies (Peto et al., 2009; Pang et al., 2019). This may be attributed to a higher proportion of HIV-positive men than women (34.8% vs 10.8%, *p* < 0.0001; **Supplementary Table S3**), although the relationship between gender and EPTB is controversial in current studies (Liu et al., 2020; Barreto-Duarte et al., 2021).

Culture is the gold standard for TB diagnosis, but culture cannot distinguish between MTB, BCG, and NTM, and its specificity is compromised (96.9% [91.3-99.2] in this study) when used in populations susceptible to NTM disease (e.g. HIV-positive patients) (Álvaro-Meca et al., 2015). Unexpectedly, the sensitivity (%65.3 [56.3-73.2] vs 56.1% [47.0-64.9], *p* = 0.16) of the smear was slightly higher than that of the culture. Compared to direct smears, centrifugally concentrated specimens can increase the sensitivity of the smear by 10-30%(Perera and Arachchi, 1999; Peterson et al., 1999), and the Auramine O staining used in this study had a higher sensitivity (66-85.9% vs 30-60%) than Ziehl-Neelsen staining (Marais et al., 2008; Laifangbam et al., 2009; Hooja et al., 2011; Runa et al., 2011; Assefa et al., 2021; Gulati et al., 2021), but the NaOH used to digest the specimens may have reduced the viability of mycobacteria or even killed mycobacteria (Mtafya et al., 2019; Stephenson et al., 2021). Auramine O staining is not able to distinguish between dead or live bacteria, but culture only detects viable bacteria, thus NaOH used in sample pre-treatment may result in lower sensitivity of culture than smear. Obtaining appropriate specimens for histological examinations was recommended for a patient with suspected EPTB (Hopewell et al., 2006; Migliori et al., 2018). In general, histopathology is highly sensitive (86%-95% reported; HE: 92.4% [85.7-96.1] and AFB: 81.7% [73.2-88.0] in this study), but not very specific (64%-92% reported; HE: 71.3% [61.0-79.7] and AFB: 73.3% [63.1-81.5] in this study), for the diagnosis of tuberculosis (Bennani et al., 2019; Shen et al., 2022; Tahseen et al., 2022).

Xpert was recommended by the World Health Organization as a rapid initial diagnostic test for tuberculosis (WHO, 2021b). For the diagnosis of EPTB, Xpert showed different performance in various types of samples (Scott et al., 2014; Kohli et al., 2021), with excellent performance in lymph node tissue and aspirates (sensitivity: 80-100%; specificity:90-100%) (Ablanedo-Terrazas et al., 2014; Scott et al., 2014; Tadesse et al., 2019), as demonstrated in this study (sensitivity: 96.6%[91.6-98.7]; specificity:100%[95.8-100]). HIV-positive patients are more likely to have comorbidities such as tumors (Lerner et al., 2020), and opportunistic infections (fungal infections, NTM infections, etc.) (Limper et al., 2017), and this may affect the specificity of detection of MTB (decreased specificity in this study: culture 5%; smear 8%; HE 23%; AFB 27%). However, Xpert maintained high sensitivity (>95%) and specificity (nearly 100%) in both HIV-positive and -negative patients, consistent with previous reports (sensitivity: >80%; specificity: 97-99%) (Horne et al., 2019; Tomaz et al., 2021). Finally, we evaluated the performance of different assays in smear-negative samples, and Xpert still had higher sensitivity (92.7% [80.6-97.5]) and specificity (100% [95.7-100]) compared to culture. This was slightly higher than the reported sensitivity (70-85%) (Bankar et al., 2018; Rakotoarivelo et al., 2018; Horne et al., 2019), perhaps due to the different choice of the reference standard (culture and Xpert were used in this study).

We found little statistical difference in the sensitivity, specificity, and predictive values of the non-biopsy samples-based Xpert compared to culture, mainly due to the small sample size. However, in agreement with previous studies, TSPOT showed a high sensitivity (70-100%) compared to culture and Xpert (Zhou et al., 2015; Li et al., 2020), predicting that TSPOT can be used as a powerful screening method for EPTB (Antel et al., 2020). Furthermore, the culture and Xpert performed better with biopsy samples than with non-biopsy samples, suggesting that biopsy is important for the accurate diagnosis of EPTB.

There are several limitations to this study. The smaller sample size of non-biopsy samples may affect the methodological comparison between non-biopsy samples and biopsy samples. The small number of samples assayed by various methods in non-biopsy samples was not conducive to evaluating the diagnostic accuracy of the method, for instance, the sensitivity of Xpert may be underestimated. In addition, we did not exclude patients with both pulmonary and extrapulmonary TB (42 cases), which may affect the comparison of assays between biopsy and non-biopsy samples.

In summary, our study compared the diagnostic accuracy of commonly used EPTB diagnostic methods across HIV status and sample types, highlighting the superiority of Xpert in different clinical settings and the critical contribution of biopsy samples in the diagnosis of EPTB. Further clinical studies evaluating the performance of the different laboratory assays in extrapulmonary samples and HIV populations are warranted to help clinicians choose the best diagnostic methods when faced with various dilemmas.

## Data availability statement

The original contributions presented in the study are included in the article/**Supplementary Material**. Further inquiries can be directed to the corresponding authors.

## Ethics statement

The studies involving human participants were reviewed and approved by Ethics Committee of Shanghai Public Health Clinical Center (2019-S030-02). Written informed consent to participate in this study was provided by the participants’ legal guardian/next of kin.

## Author contributions

Project concept conceived (X-YF and HZ), experiments performed (J-CX and W-FG), sampling (XM and XS), data analysis (J-CX, XM, W-FG, X-YF, and HZ), and paper writing (J-CX and X-YF). All authors contributed to the article and approved the submitted version.
