# Supplementary material for: Diagnosis of extrapulmonary tuberculosis by ultrasound-guided biopsy: A retrospective comparison study
Source: Front Cell Infect Microbiol. 2023 Mar 22;13:1154939. doi: 10.3389/fcimb.2023.1154939 (PMC10075254; doi:10.3389/fcimb.2023.1154939)
Supplement: Supplementary file 1 [file Table_1.docx]

**Supplementary Table S1. Head-to-head comparison of test accuracy in biopsy samples, by HIV status**

|  |  | Sensitivity | Specificity | PPV | NPV |
| --- | --- | --- | --- | --- | --- |
| HIV  negative | Culture | 50/94 (53.2; 43.2-63.0) | 65/66 (98.5; 91.9-99.9) | 50/51 (98.0; 89.7-99.9) | 65/109 (59.6; 50.3-68.4) |
|  | HIV negative *vs* HIV positive | 0.17 | 0.50 | 0.28 | **0.012** |
|  | Smear | 62/98 (63.3; 53.4-72.1) | 67/68 (98.5; 92.1-99.9) | 62/63 (98.4; 91.5-99.9) | 67/103 (65.1; 55.5-73.6) |
|  | HIV negative *vs* HIV positive | 0.32 | 0.17 | **0.033** | **0.031** |
|  | Xpert | 95/98 (96.9; 91.4-99.2) | 66/66 (100; 94.5-100) | 95/95 (100; 96.1-100) | 66/69 (95.7; 88.0-98.8) |
|  | HIV negative *vs* HIV positive | 0.66 | > 0.99 | > 0.99 | > 0.99 |
|  | HE | 79/86 (91.9; 84.1-96.0) | 49/63 (77.8; 66.1-86.3) | 79/93 (85.0; 76.3-90.8) | 49/56 (87.5; 76.4-93.8) |
|  | HIV negative *vs* HIV positive | 0.67 | 0.056 | **0.016** | 0.57 |
|  | AFB | 68/85 (80.0; 70.3-87.1) | 49/60 (81.7; 70.1-89.4) | 68/79 (86.1; 76.8-92.0) | 49/66 (74.2; 62.6-83.3) |
|  | HIV negative *vs* HIV positive | 0.33 | **0.016** | **0.0047** | 0.26 |
| HIV  positive | Culture | 14/20 (70.0; 48.1-85.5) | 29/31 (93.6; 79.3-98.9) | 14/16 (87.5; 64.0-97.8) | 29/35 (82.9; 67.3-91.9) |
|  | Smear | 15/20 (75.0; 53.1-88.8) | 28/31 (90.3; 75.1-96.7) | 15/18 (83.3; 60.8-94.2) | 28/33 (84.9; 69.1-93.4) |
|  | Xpert | 19/20 (95.0; 76.4-99.7) | 22/22 (100; 85.1-100) | 19/19 (100; 83.2-100) | 22/23 (95.7; 79.0-99.8) |
|  | HE | 18/19 (94.7; 75.4-99.7) | 13/24 (54.2; 35.1-72.1) | 18/29 (62.1; 44.0-77.3) | 13/14 (92.7; 68.5-99.6) |
|  | AFB | 17/19 (89.5; 68.6-98.1) | 14/26 (53.9; 35.5-71.2) | 17/29 (58.6; 40.7-74.5) | 14/16 (87.5; 64.0-97.8) |

**Supplementary Table S2 Diagnostic accuracy of culture and Xpert in paired biopsy samples (BS) and non-biopsy samples (NBS)**

|  |  | Sensitivity | Specificity | PPV | NPV |
| --- | --- | --- | --- | --- | --- |
| Biopsy sample | Culture | 14/25(56.0; 37.1-73.3) | 28/28(100.0; 87.9-100.0) | 14/14(100.0; 78.5-100.0) | 28/39(71.8; 56.2-83.5) |
|  | BS vs NBS | 0.19 | >0.99 | >0.99 | 0.64 |
|  | Xpert | 25/25(100.0; 86.7-100.0) | 24/24(100.0; 86.2-100.0) | 25/25(100.0; 86.7-100.0) | 24/24(100.0; 86.2-100.0) |
|  | BS vs NBS | 0.022 | >0.99 | >0.99 | 0.032 |
| Non-biopsy sample | Culture | 6/17(35.3; 17.3-58.7) | 22/22(100.0; 85.1-100.0) | 6/6(100.0; 61.0-100.0) | 22/33(66.7; 49.6-80.3) |
|  | Xpert | 3/5(60.0; 23.1-92.9) | 9/9(100.0; 70.1-100.0) | 3/3(100.0; 43.9-100.0) | 9/11(81.8; 52.3-96.8) |

**Supplementary Table S3 Gender composition of HIV-positive and negative patients**

|  | HIV positive | HIV negative |
| --- | --- | --- |
| Female | 11（10.8%） | 91（89.2%） |
| Male | 40（34.8%） | 75（65.2%） |
